# *Mycoplasma genitalium*: An Emerging Cause of Sexually Transmitted Disease in Women

**DOI:** 10.1371/journal.ppat.1001324

**Published:** 2011-05-26

**Authors:** Chris L. McGowin, Colin Anderson-Smits

**Affiliations:** 1 Department of Medicine, Section of Infectious Diseases, Louisiana State University Health Sciences Center, New Orleans, Louisiana, United States of America; 2 Department of Epidemiology, Tulane University School of Public Health and Tropical Medicine, New Orleans, Louisiana, United States of America; University of California San Diego, United States of America

## Abstract

*Mycoplasma genitalium* is an emerging sexually transmitted pathogen implicated in urethritis in men and several inflammatory reproductive tract syndromes in women including cervicitis, pelvic inflammatory disease (PID), and infertility. This comprehensive review critically examines epidemiologic studies of *M. genitalium* infections in women with the goal of assessing the associations with reproductive tract disease and enhancing awareness of this emerging pathogen. Over 27,000 women from 48 published reports have been screened for *M. genitalium* urogenital infection in high- or low-risk populations worldwide with an overall prevalence of 7.3% and 2.0%, respectively. *M. genitalium* was present in the general population at rates between those of *Chlamydia trachomatis* and *Neisseria gonorrhoeae*. Considering more than 20 studies of lower tract inflammation, *M. genitalium* has been positively associated with urethritis, vaginal discharge, and microscopic signs of cervicitis and/or mucopurulent cervical discharge in seven of 14 studies. A consistent case definition of cervicitis is lacking and will be required for comprehensive understanding of these associations. Importantly, evidence for *M. genitalium* PID and infertility are quite convincing and indicate that a significant proportion of upper tract inflammation may be attributed to this elusive pathogen. Collectively, *M. genitalium* is highly prevalent in high- and low-risk populations, and should be considered an etiologic agent of select reproductive tract disease syndromes in women.

## Introduction

An estimated 340 million new curable cases of sexually transmitted infections (STIs) are acquired annually throughout the world [1], making these infections an important public health and economic concern. *Mycoplasma genitalium* is an emerging cause of STIs in the United States [2] and has been implicated in urogenital infections of men and women around the world. More than 25 years after its initial isolation from men with non-gonococcal urethritis (NGU; [3]), *M. genitalium* is now recognized as an independent etiologic agent of acute and persistent male NGU and is responsible for approximately 20%–35% of non-chlamydial NGU cases [4], [5]. Implicating this organism in male urogenital disease was a significant advancement in our knowledge of STIs, but it has been less clear whether *M. genitalium* is also a cause of inflammatory reproductive tract disease in women. This comprehensive literature review (PubMed database; MeSH “*Mycoplasma genitalium*” with no restrictions on publication year) addresses the overall population prevalence and associations of *M. genitalium* with inflammatory syndromes of the female reproductive tract.

## Epidemiology and Prevalence of *M. genitalium* Infections

After the initial isolation in 1980 [3], few epidemiologic studies of *M. genitalium* infection were undertaken largely because of difficulties in cultivation of this fastidious organism. Some 10 years later, the polymerase chain reaction (PCR) was first employed for detection of *M. genitalium* in patient specimens [6], [7], thereby facilitating larger investigations of prevalence and associations with urogenital disease. Using such molecular methods, sexual transmission of the organism has been suggested by high concordance rates among sexual partners [8]–[15] and documented specifically in infected couples with concordant *M. genitalium* genotypes [11], [15]. In addition, sexual transmission of *M. genitalium* infection can be inferred from increased prevalence values in cohorts reporting sexual intercourse and the association with number of sex partners [2], [16]. For the purpose of this review, high-risk individuals were defined as those attending an STI clinic, those enrolled in a study where inclusion criteria included signs of urogenital disease, patients presenting to family planning clinics for termination of pregnancy, or those individuals classified as sex workers. Low-risk enrollees were those not attending an STI clinic, fertility clinic attendees, those chosen randomly from an otherwise healthy population, and all women enrolled in studies of adverse pregnancy outcomes.

Considering 27,272 women from 40 independent studies, among women from low-risk populations (*n* = 8,434; [2], [14], [16]–[24]), the prevalence of *M. genitalium* infection was 2.0% with most cohorts between <1%–5% (Table S1). In three studies of low-risk individuals where enrollees were randomly selected from community or population-based survey populations ([2], [16], [19]; *n* = 4,075), the prevalence was also 2%. Among these was the US National Longitudinal Study of Adolescent Health, which showed the genital prevalence of *M. genitalium* to be approximately 1%, between those of *Neisseria gonorrhoeae* (0.4%) and *C. trachomatis* (4.2%), respectively [2]. Thus, *M. genitalium* prevalence in the general population is on par with other sexually transmitted pathogens of significant public health concern.

Using the above definition of high-risk populations, 18,838 women have been tested for *M. genitalium* urogenital infection [6]–[8], [10], [13], [25]–[56]with a substantially higher prevalence than low-risk groups (7.3%; Table S1). Among studies of high-risk individuals, the population prevalence values ranged from 0% to 42% and can be explained by several factors, including the clinical setting, specificity of the employed NAAT assay, participant enrollment criteria (e.g., specific symptoms or signs), geographic location of study site, high-risk behavior (e.g., commercial sex workers), co-infection with other STIs, and the reporting of point or cumulative, multi-sampling values. Importantly, we considered only point prevalence values in the overall prevalence calculations because cumulative, multi-sampling values were not directly comparable. In conclusion, considering sexual transmission and the high prevalence worldwide, the public health significance of *M. genitalium* infections in women is potentially very large.

## Clinical Correlates with Lower Urogenital Tract Inflammation in Women

### Vaginal Discharge

In more than 20 independent clinical studies, *M. genitalium* has been evaluated as a cause of inflammatory lower genital tract syndromes including urethritis, cervicitis, and vaginal discharge. From 2002 to 2010, six studies addressed the relationship between *M. genitalium* infection and vaginal discharge (*n* = 3,059; Table 1) with significant associations observed in three [14], [43], [51]. Vaginal discharge was measured either as a symptom or sign among the cited studies and the criteria varied from any sign of discharge to defined pathologic symptoms such as discharge characterized as heavy, yellow, or green-gray with mucous-like or creamy consistency (see Table 1 for specific diagnostic criteria for each study). No clear trend was evident as to whether signs or symptoms were better predictors of *M. genitalium* infection, as only two studies used vaginal symptoms in their diagnostic criteria.

**Table 1:** Characteristics of published studies evaluating the associations of *M. genitalium* with vaginal discharge or urethritis.

Reference, Clinical Setting, and Specific Criteria for Enrollment	No. Tested	MG Prevalence or Incidence (%)	Diagnostic Criteria for Vaginal or Urethral Signs/Symptoms	MG Prevalence in Cases, Controls (%)^1^	Relative Risk Measure Expressed as OR or HR (95% CI); Adjusted Variable(s) in Multivariate Analyses Are Indicated If Applicable
**VAGINAL DISCHARGE**					
**Cohen, 2007** ([33]; PHC, CSW, Kenya)	255	16.0^2^	Sign of pathologic vaginal discharge	Not calculable from presented data	0.84 (0.40–1.78)
**Huppert, 2008** ([41]; THC or ER, genital signs or high-risk behavior, US)	331	22.4	Sign of pathologic vaginal discharge	26/137 (19), 45/181 (24.9)	Sign of pathologic discharge 0.71 (0.41–1.22)
**Korte, 2006** ([43]; PHC, STI at enrollment, US)	674	42.0^3^	Symptom of vaginal discharge or pathologic vaginal discharge (heavy, yellow, green-gray, or consistency was mucous-like or creamy)	Vaginal discharge 30/33 (90.1), 199/300 (66.3)^3^; Pathologic vaginal discharge 28/31 (90.3), 199/300 (66.3)^3^	Vaginal discharge adjusted for A, PS, NG, CT, TV, CA, BV 4.8 (1.4–16.5); Pathologic vaginal discharge adjusted for A, PS, NG, CT, TV, CA, BV 3.5 (1.2–10.3)
**Pepin, 2005** ([51]; STI clinic, CSW, Benin/Ghana)	826	26.3	Sign of vaginal discharge	53/122 (43.4), 164/505 (32.5)^4^	1.6 (1.0–2.45)
**Tosh, 2007** ([14]; Primary health care clinic, US)	383	13.6^5^	Sign of vaginal discharge	Vaginal discharge 12/56 (21.4), 25/327 (7.6)^6^	Vaginal discharge 3.30 (1.54–7.03)
**Thurman, 2010** ([13]; PHC, STI at enrollment, US)	590	9.2^5^	Symptom of any vaginal discharge or pathologic vaginal discharge (heavy, yellow, green-gray, or consistency was mucous-like or creamy)	15/203 (7.4), 55/609 (9.0)^7^; 10/174 (5.7), 60/638 (9.4)^7^	Vaginal discharge 0.80 (0.45–1.45); Pathologic vaginal discharge 0.59 (0.30–1.16)
**URETHRITIS**					
**Anagrius, 2005** ([8]; STI clinic, Sweden)	445	6.3	US4 pmn/hpf	11/129 (8.5), 6/227 (2.6)^8^	2.3 (0.96–5.64)
**Falk, 2005** ([10]; STI clinic or cancer screen controls, Sweden)	520	5.0^6^	US>4 pmn/hpf	2/47 (4.3), 20/410 (4.9)^6^^,^ 8	0.87 (0.20–3.83)
**Hogdahl, 2007** ([40]; STI clinic, Sweden)	417	6.5	US>4 pmn/hpf	12/26 (46.2), 110/377 (29.2)	2.08 (0.93–4.64)
**Moi, 2009** ([49]; STI clinic, genital signs, or high-risk behavior, Norway)	7604^6^	4.5^6^	US>10 pmn/hpf	64/187 (34), 1452/6603 (22)^6^^,^ 9	2.1 (1.5–2.9)

1 Cases are those individuals with defined signs or symptoms; controls are individuals without signs or symptoms.

2 *M. genitalium* prevalence and OR calculated at enrollment.

3 Cumulative, multi-sampling prevalence over a 5-y study that included *M. genitalium* PCR- and culture-positive individuals; only PCR-positive individuals considered for prevalence in cases vs. controls and OR calculations.

4 Excluded women co-infected with NG, CT, and TV.

5 Cumulative, multi-sampling prevalence.

6 Excluded patients co-infected with *M. genitalium* and CT.

7 OR calculated from symptoms reported at each sampling and excluded patients co-infected with *M. genitalium* and CT or NG.

8 Excluded patients with concurrent cervicitis.

9 For OR calculation and prevalence in cases versus controls, number of *M. genitalium* infections represented FVU-positive samples; overall prevalence for entire study population determined from FVU and endocervical swabs.

MG, *Mycoplasma genitalium*; OR, odds ratio; RR, relative risk; HR, hazard ratio; pmn/hpf, polymorphonuclear leukocytes per high-power microscope field; STI, sexually transmitted infection; PHC, public health clinic; THC, teen health center; ER, emergency room; WH, women's health; FVU, first void urine; US, urethral swab; A, age; CT, *Chlamydia trachomatis* status; NG, *Neisseria gonorrhoeae* status; TV, *Trichomonas vaginalis* status; PS, pregnancy status, CA, *Candida albicans* status, BV, bacterial vaginosis status.

Etiologies of vaginal discharge are extremely diverse, can be either microbial or non-microbial, may be normal or abnormal, and can be attributed to inflammation in other parts of the reproductive tract (reviewed in [57]). Only one study adjusted for the presence of bacterial vaginosis (BV; [43]) and, despite a high rate of BV in the cohort, demonstrated significant associations of *M. genitalium* with vaginal discharge. However, evaluation of a similar population 4 years later did not reproduce the finding [13]. Further, the case definition of pathologic discharge was variable between studies and, most importantly, patient-reported symptoms are a highly subjective measure. Therefore, the disparity among studies is not surprising. Future studies with defined and/or quantitative signs that control for co-infection with other STIs and concurrent inflammatory syndromes (e.g., bacterial vaginosis) will be necessary to determine whether *M. genitalium* is independently associated with vaginal discharge.

### Urethritis

Considering only microscopic signs of urethral inflammation (>4–5 or >10 polymorphonuclear leukocytes per high-powered microscope field [PMNL/hpf]), positive associations with *M. genitalium* infection were found in three of four studies [8], [40], [49]. One study, the largest of Scandinavian women ([49]; *n* = 7,604; Table 1), found a significant association between *M. genitalium* and microscopic urethritis. Three other Scandinavian studies of urethritis failed to show a significant association with *M. genitalium* infection even when patients co-infected with *C. trachomatis* [10] were removed or after adjusting for concurrent cervicitis [8], [9]. However, Anagrius and colleagues showed a significant association with microscopic signs of urethritis and/or cervicitis in Swedish women [8]. This study exemplifies that exclusion of women with concurrent cervicitis or other inflammatory syndromes is important because inflammation from other sites may contaminate the urethra leading to a false diagnosis of urethritis. Two of the studies that failed to show a significant association between *M. genitalium* and urethritis, but did control for concurrent cervicitis [8], [40], showed a strong trend towards association with lower bounds of their respective 95% CIs close to the null. Importantly, *M. genitalium* is a recognized cause of sexually acquired acute and persistent urethritis [4], [5] in men. However, considering the disparate results of the cited studies, we cannot conclusively implicate *M. genitalium* as a cause of female urethritis. Additional investigations of *M. genitalium* urethritis are warranted, especially in populations outside of Scandinavia.

### Cervicitis

Cervicitis, often termed mucopurulent cervicitis [58], is characterized by the presence of clinical signs such as mucopurulent discharge, friability at the external os (easily induced bleeding), elevated counts of PMNL detected by Gram staining of endocervical swab material, or a combination of these signs [58]. However, there is no generally accepted case definition of cervicitis. Among epidemiologic studies of cervicitis in high- and low-risk populations (Table 2; *n* = 13,000 women), *M. genitalium* has been positively associated with cervical inflammation in all studies where microscopic signs were considered independent of non-microscopic signs [8], [10], [30], [40], [47], [49]. Of these, only two studies showed significant correlations [10], [49]. Considering non-microscopic criteria (see study by Pepin et al. [51] for diversity of non-microscopic signs), cervical discharge was the most consistently measured among the retained studies. Four of eight studies showed positive associations between cervical discharge and *M. genitalium* infection, all of which were significant relative to women without this sign (Table 2; [25], [36], [47], [51]).

**Table 2:** Characteristics of published studies evaluating the associations of *M. genitalium* with cervicitis.

Reference, Clinical Setting, and Specific Criteria for Enrollment	No. Tested	MG Prevalence or Incidence (%)	Diagnostic Criteria for Cervicitis Cases	MG Prevalence in Cases, Controls (%)^1^	Relative Risk Measure Expressed as OR or HR (95% CI); Adjusted Variable(s) in Multivariate Analyses Are Indicated If Applicable
**Anagrius, 2005** ([8]; STI clinic, Sweden)	445	6.3	Microscopic signs (>30 pmn/hpf)	4/30 (13.3), 22/327 (6.7)^2^	2.13 (0.68–6.66)
**Arraiz, 2008** ([25]; Private Ob/Gyn clinic; genitourinary signs, Venezuela)	172	7.6^3^	Genitourinary symptoms including mucopurulent cervical discharge	9/74 (12.2), 4/98 (4.1)^3^	Any symptom 3.2 (1–11)
**Casin, 2002** ([30]; STI clinic, vaginal discharge, France)	170	38.2^4^	Microscopic signs (>10 pmn/hpf) or erythematous cervix or mucopurulent cervical discharge	Erythematous cervix 26/77 (34), 38/91 (42); Mucopurulent cervical discharge 54/145 (37), 10/23 (43); >10 pmn/hpf 42/99 (42), 23/71 (32)	Erythematous cervix 0.71 (0.4–1.3); Mucopurulent cervical discharge 0.77 (0.3–1.9); >10 pmn/hpf 1.54 (0.8–2.9)
**Cohen, 2007** ([33]; PHC, CSW, Kenya)	255	16.0	Signs of cervical erythema and mucopurulent discharge	Not calculable from presented data	Both signs 0.6 (0.2–1.9)
**Falk, 2005** ([10]; STI clinic or cancer screen controls, Sweden)	520	5.0^5^	Microscopic signs (>pmn/hpf than epithelial cells)	9/30 (30.0), 13/431 (3.0)^2^	13.78 (5.30–35.86)
**Gaydos, 2009** ([36]; STI clinic, US)	322	19.3	Signs of cervical discharge or cervical friability	38/133 (28.6), 24/191 (12.6)	Either sign, crude 2.8 (1.6–4.9); Either sign, adjusted for CT, NG, TV 2.5 (1.4–4.5); Either sign, adjusted for CT, NG, TV, A, BV, RC 2.4 (1.3–4.4)
**Hogdahl, 2007** ([40]; STI clinic, Sweden)	417	6.5	Microscopic signs (>30 pmn/hpf)	9/110 (8.2), 17/293 (5.8)	1.45 (0.62–3.35)
**Huppert, 2008** ([41]; THC or ER, genital signs or high-risk behavior, US)	331	22.3	Signs of cervical discharge (yellow or purulent), cervical friability, or cervical motion tenderness	Cervical discharge 7/47 (14.9), 64/271 (23.6); Friable cervix 11/52 (21.2), 60/266 (22.6); Discharge or friable cervix 18/87 (19.5), 54/231 (23.4); Cervical motion tenderness 6/39 (15.4), 65/279 (23.3)	Cervical discharge 0.6 (0.2–1.3); Friable cervix 0.9 (0.4–1.9); Discharge or friable cervix 0.8 (0.4–1.5); Cervical motion tenderness 0.6 (0.2–1.5)
**Korte, 2006** ([43]; PHC, STI at enrollment, US)	674	42.0^6^	Sign of cervical mucopus	7/10 (70), 250/364 (68.7)^6^	Adjusted for A, PS, NG, CT, TV, CA, BV 0.65 (0.15–2.9)
**Manhart, 2003** ([46]; STI clinic, US)	719	7.0	Microscopic signs (>30 pmn/hpf) or visible yellow mucopus	Either sign 24/215 (11.2), 26/504 (5.2)	Either sign, adjusted for A, PMC, CT, NG 3.1 (1.46–6.75)
**Manhart, 2008** ([47]; STI clinic, HIV-positive, 72% CSW, Kenya)	303	17.2	Microscopic signs (>30 pmn) or cervical discharge (cloudy/white, yellow/green, brown, or bloody) or easily induced cervical bleeding	Cervical discharge 23/90 (25.6), 29/213 (13.6); >30 pmn/hpf 17/85 (20), 35/218 (16.1); Easily induced cervical bleeding 9/52 (17.3), 43/251 (17.1)	Cervical discharge 2.2 (1.2–4.0); >30 pmn/hpf 1.3 (0.7–2.5); Easily induced cervical bleeding 1 (0.5–2.2)
**Moi, 2009** ([49]; STI clinic, genital signs or high-risk behavior, Norway)	7604^5^	4.5^5^	Microscopic signs (>30 pmn/hpf)	128/3007 (4.3), 124/3643 (3.4)^5^	1.3 (1.0–1.6)
**Pepin, 2005** ([51]; STI clinic, CSW, Benin/Ghana)	826	26.3	Signs of cervical discharge, pus on swab, bleeding after sampling, edema and erythema, or cervical motion tenderness	Cervical discharge 38/70 (54.3), 179/555 (32.3)^7^; Pus on swab 34/62 (54.8), 172/535 (32.1)^7^; Bleeding after sampling 26/39 (66.6), 190/586 (32.4)^7^; Edema and erythema 40/76 (52.6), 175/547 (32)^7^; Cervical motion tenderness 39/82 (47.6), 178/544 (32.7)^7^	Cervical discharge, adjusted for CT, NG, TV 1.6 (1–2.5); Pus on swab, adjusted for CT, NG, TV 1.6 (1–2.7); Bleeding after sampling, adjusted for A, CT, NG, TV 1.8 (1–3.1); Edema and erythema, adjusted for A, CT, NG, TV 1.6 (1–2.5); Cervical motion tenderness, adjusted for A, CT, NG, TV 1.3 (0.9–2)
**Uno, 1997** ([56]; Hospital Ob/Gyn or pregnant control patients, Japan)	200	4.5	Microscopic signs (>20 pmn/hpf) or cervical discharge	5/57 (7.8), 0/79 (0)^8^	Either sign, 16.65 (0.90–307.62)

1 Cases are those individuals with defined signs or symptoms; controls are individuals without signs or symptoms.

2 Excluded patients with concurrent urethritis and those co-infected with *M. genitalium* and CT.

3 Population prevalence and prevalence of *M. genitalium* in cases vs. controls excluded patients with human papilloma virus and CT.

4 Each enrolled patient sampled at 2–3 sites; prevalence calculated from positive result at any site.

5 Exluded women co-infected with *M. genitalium* and CT.

6 Cumulative, multi-sampling prevalence over a 5-y study that included *M. genitalium* PCR- and culture-positive individuals; only PCR-positive individuals considered for prevalence in cases vs. controls and OR calculations.

7 Excluded women co-infected with *M. genitalium* and NG, CT, or TV.

8 Excluded women with CT and women co-infected with *M. genitalium* and CT.

Two studies have addressed whether microscopic or non-microscopic signs are better predictors of *M. genitalium* cervicitis within the same patient population. Casin et al. found no significant associations using either cervical discharge or microscopic signs (>10 pmn/hpf) [30], but all women in this study had vaginal discharge. However, Manhart and colleagues found no significant association between *M. genitalium* infection and cervicitis defined by >30 PMNL/hpf, but statistical significance was observed with abnormal cervical discharge ([47]; Table 2). Further, considering all studies of *M. genitalium* cervicitis, those where a high threshold of microscopic cervicitis (>20 or >30 PMNL/hpf, or more PMNL than epithelial cells) was employed, only three of seven studies showed a significant correlation between microscopic signs and *M. genitalium* infection ([10], [46], [49]; Table 2). This suggests that a high microscopic threshold of inflammation is not a more specific sign of *M. genitalium* cervicitis and might also fail to detect less severe inflammation. Collectively, it is clear that discrepancies among these studies can be attributed to the variable case definition of cervicitis and studies with uniform criteria will be required to address which sign(s) best predict *M. genitalium* cervicitis.

*C. trachomatis* is a common cause of cervicitis and a potentially confounding variable for implicating *M. genitalium* as an independent etiologic agent. Where possible, we excluded subjects with *C. trachomatis* co-infection for all OR calculations (see Table 2). Six of nine studies where *C. trachomatis* co-infection was either excluded or adjusted for in multivariate analyses found significant associations between *M. genitalium* and cervicitis [10], [25], [36], [46], [49], [51], whereas only a single study [47] showed significant associations without this adjustment. Studies controlling for *N. gonorrhoeae* infection are lacking. Despite these differences, several clinical investigations of urogenital disease in women indeed indicate that *M. genitalium* should be considered an independent risk factor for cervicitis, particularly when urogenital specimens are negative for other known pathogens. Importantly, this magnitude of increased risk is similar to those of other known causes of cervicitis, including *C. trachomatis* and *N. gonorrhoeae* [46].

## Upper Reproductive Tract Infection by *M. genitalium*

### Pelvic Inflammatory Disease

Following sexual transmission, cervical passage of *M. genitalium* could result in ascending infection of the endometrium or further to the fallopian tubes leading to tubal inflammation and infertility. *M. genitalium* was first suspected as a cause of pelvic inflammatory disease (PID) in 1984 [59]. Since, five PCR-based studies have found a positive association of *M. genitalium* with clinical PID from geographically diverse populations around the world ([27], [31], [39], [54], [56]; Table 3). In the first of these, of 58 Kenyan women with histologically confirmed endometritis, *M. genitalium* was found significantly more often in women with endometritis compared to women without this condition (16% versus 2%; [31]). Similarly, in a sub-study from the US PID Evaluation of Clinical Health cohort, women with *M. genitalium* were three times more likely to have endometritis at enrollment compared to women without *M. genitalium* [39].

**Table 3:** Characteristics of published studies evaluating the associations of *M. genitalium* with pelvic inflammatory disease.

Reference, Clinical Setting, and Specific Criteria for Enrollment	No. Tested	MG Prevalence or Incidence (%)	Diagnostic Criteria for PID or Endometritis Cases	MG Prevalence in Cases, Controls (%)^1^	Relative Risk Measure Expressed as OR or HR (95% CI); Adjusted Variable(s) in Multivariate Analyses Are Indicated If Applicable
**Bjartling, 2010** ([27]; Hospital Ob/Gyn, requesting TOP, Sweden)	2079	2.5	Lower abdominal pain, cervical, uterine, or adnexal tenderness together with one of pathological vaginal wet smear or yellow endocervical pus, elevated C-reactive protein >8, or fever >38°C	6/49 (12.2), 4/168 (2.4)	PID adjusted for A, CT 6.29 (1.56–25.2)
**Cohen, 2002** ([31]; STI clinic, pelvic pain >14 d, Kenya)	115	8.7	At least 1 plasma cell per hpf of endometrial stroma (endometritis)	9/58 (15.5), 1/57 (1.8)	10.29 (1.26–84.14)
**Cohen, 2007** ([33]; PHC, CSW, Kenya)	255	15.7^2^	Clinical PID not defined	27/109 (24.7), 50/135 (37)	PID since enrollment 0.70 (0.43–1.13)
**Haggerty, 2008** ([39]; ER, Ob/Gyn, STI, or primary health clinic, clinically suspected PID or endometritis, US)	586	15.0^3^	At least 5 neutrophils in the endometrial surface epithelium in the absence of menstrual endometrium and/or at least two plasma cells in the endometrial stroma (endometritis)	43/240 (17.9), 20/262 (7.6)^3^	Endometritis at enrollment, adjusted for A, R, PT, IC 3.0 (1.5–6.1)
**Jurstrand, 2007** ([42]; Ob/Gyn, PID or ectopic pregnancy, Sweden)	521	16.1^4^	Pain in lower abdomen for <3 wks, palpable adnexal mass and/or motion tenderness, fever >38°C and objective signs of lower tract infection	33/193 (17), 36/246 (14.6)^4^	Adjusted for A, CT 1.0 (0.6–1.7)
**Simms, 2003** ([54]; STI clinic or Ob/Gyn, primary health clinic for controls, UK)	82	7.3	Lower abdominal pain, adnexal tenderness, and cervical/uterine motion tenderness	5/44 (11.4), 0/37 (0)^5^	10.43 (0.56–195.4)
**Uno, 1997** ([56]; Hospital Ob/Gyn or pregnant control patients, Japan)	200	4.5	Abdominal pain, adnexal tenderness, fever >37°C, and leukocytosis	PID 2/49 (3.8), 0/79 (0)^6^	8.47 (0.17–199.06)

1 Cases are those individuals with defined signs or symptoms; controls are individuals without signs or symptoms.

2 *M. genitalium* prevalence calculated at enrollment; PID HR calculated from incident infections in prospective study.

3 *M. genitalium* prevalence and association with endometritis calculated from infection status and signs at enrollment.

4 *M. genitalium* prevalence and association with ectopic pregnancy were evaluated using a serological assay rather than with a NAAT.

5 Excluded patients co-infected with *M. genitalium* and CT.

6 Excluded women with CT and women co-infected with *M. genitalium* and CT.

MG, *Mycoplasma genitalium*; OR, odds ratio; RR, relative risk; HR, hazard ratio; hpf, high-power microscope field; Ob/Gyn, obstetrics and gynecology; STI, sexually transmitted infection; PID, pelvic inflammatory disease; ER, emergency room; CSW, commercial sex workers; TOP, termination of pregnancy; PHC, public health clinic; PT, self reported partner treatment; A, age; CT, *Chlamydia trachomatis* status; R, race; IC, intercourse between enrollment and 30-d visit.

In two cross-sectional studies where the endometrium was sampled directly to measure the associations of current infection and upper tract disease [31], [33], [39], *M. genitalium* was associated significantly with endometritis [31], [39]. In contrast, one prospective study of commercial sex workers in Kenya failed to find an association of *M. genitalium* infection with PID [33] over 36 months. Considering the persistent nature of *M. genitalium*, as with other STIs, it is possible that the follow-up period and high percentage of loss to follow-up was not adequate to detect incident PID. Importantly, clinical diagnosis of PID includes several variable signs (see Table 3) that often do not correlate with laparoscopic findings [54]; this undoubtedly contributes to variability among PID studies and could impact the associations with *M. genitalium* infection. Further, no clear trend was observed when comparing studies that removed co-infections with *C. trachomatis* or controlled for co-infections in multivariate analyses. Overall, *M. genitalium* has been associated with microscopic endometritis and PID, and confirmatory studies are clearly necessary to establish an independent role and investigate the mechanisms for upper reproductive tract inflammation.

### Pregnancy-Related Complications and Infertility

PID can be a pre-cursor to several significant upper tract complications, including ectopic pregnancy, chronic pelvic pain and tubal factor infertility [60]. No association between *M. genitalium* and ectopic pregnancy was observed in a single study using serological testing for *M. genitalium* exposure ([42]; Table 4). Considering other adverse pregnancy outcomes including preterm birth, spontaneous abortion or miscarriage, stillbirth, and small for gestational age, of five independent studies ([20], [21], [42], [44], [53]; Table 4), two studies have indeed shown an independent association of *M. genitalium* with preterm birth [18], [20], but no other syndromes have been linked to this infection.

**Table 4:** Characteristics of published studies evaluating the associations of *M. genitalium* with pregnancy-related complications or infertility.

Reference, Clinical Setting, and Specific Criteria for Enrollment	No. Tested	MG Prevalence or Incidence (%)	Diagnostic Criteria for Cases of Pregnancy-Related Complications	MG Prevalence in Cases, Controls (%)^1^	Relative Risk Measure Expressed as OR or HR (95% CI); Adjusted Variable(s) in Multivariate Analyses Are Indicated If Applicable
**PREGNANCY-RELATED COMPLICATIONS**					
**Edwards, 2006** ([18]; Hospital Ob/Gyn, signs/symptoms of preterm labor, US)	134	20.2	Preterm birth, delivery at <37 wks gestation	Not calculable from presented data	Adjusted for anaerobic bacteria, *Gardnerella* and other mollicutes 3.48 (1.41–8.57)
**Hitti, 2010** ([20]; Ob/Gyn patients delivering preterm or controls at term, Peru)	1328	3.1	Preterm birth, delivery at 20–36 wks gestation; Control women delivered ≥37 wks	29/661 (4.4), 12/667 (1.7)	Preterm birth adjusted for MA, CS, STB, TG, PTB 2.5 (1.2–5.0)
**Jurstrand, 2007** ([42]; Hospital Ob/Gyn, ectopic pregnancy, Sweden)	521	16.1^2^	Clinical diagnosis of ectopic pregnancy	15/82 (18.2), 36/246 (15)	Ectopic pregnancy adjusted for A, CT 1.0 (0.5–2.0)
**Labbe, 2002** ([44]; Hospital Ob/Gyn, preterm or controls at term, Guinea-Bassau)	1014	6.2	Preterm birth, delivery less than 37 wks gestation; Stillbirth, stillborn delivery ≥20 wks gestation; Abortion, spontaneous abortion <20 wks gestation; Small for gestational age, birth weight <2500 g	Premature delivery 16/199 (8), 36/600 (6); Stillbirth 8/125 (6.4), 36/600 (6); Spontaneous abortion 2/53 (3.8), 36/600 (6); Small for gestational age 1/37 (2.7), 36/600 (6)	Premature delivery 1.37 (0.69–2.60); Stillbirth 1.07 (0.42–2.42); Spontaneous abortion 0.61 (0.07–2.51); Small for gestational age 0.44 (0.01–2.75)
**Oakeshott, 2004** ([21]; Pregnant primary health or family planning attendees <10 wks gestation, UK)	915	0.7	Miscarriage, pregnancy loss at <16 wks gestation; Preterm birth, delivery at <37 wks gestation	Miscarriage 1/92 (1.1), 5/802 (0.6); Preterm birth 0/39 (0), 3/660 (0.5)	Miscarriage 1.7 (0.2–15); Preterm birth 2.38 (0.12–46.8)
**Short, 2010** ([53]; Pregnant ER patients <22 wks gestation, US)	216	5.6	Spontaneous abortion, a non-induced pregnancy loss before 22 wks of gestation	3/82 (3.7), 9/134 (6.7)	Spontaneous abortion adjusted for A, PSA, CS, GA 0.9 (0.2–3.8)
**INFERTILITY**					
**Clausen, 2001** ([17]; IVF clinic, Denmark)	308	13.0^2^	Laparoscopically confirmed tubal occlusion	29/132 (22), 11/176 (6.3)^2^	Tubal factor infertility 3.8 (1.7–9.4)
**Grzesko, 2009** ([37]; Infertile Ob/Gyn patients and fertile controls, Poland)	74	14.9	Primary infertility of unknown etiologies at enrollment	All infertile patients 10/51 (19.6), 1/23 (4.4); Subset of infertile women with idiopathic infertility 7/24 (29.2), 1/23 (4.4)	All infertile patients 5.37 (0.46–44.72); Subset of infertile women with idiopathic infertility after laparoscopy 9.06 (1.02–80.89)
**Svenstrup, 2008** ([24]; Fertility clinic, Denmark)	210	0^3^	Laparoscopically or culdoscopy-confirmed tubal occlusion	5/25 (20), 10/192 (5.2)^2^^,^ 4	Tubal factor infertility adjusted for A, CTS 4.5 (1.3–15.2)

MG, *Mycoplasma genitalium*; OR, odds ratio; RR, relative risk; HR, hazard ratio; NAAT, nucleic acid amplification test; Ob/Gyn, obstetrics and gynecology; ER, emergency room; PID, pelvic inflammatory disease; IVF, in vitro fertilization; A, age; CT, *Chlamydia trachomatis* status; MA, maternal age; CS, cigarette smoking; STB, second trimester bleeding; TG, twin gestation; PTB, prior preterm birth; PSA, previous spontaneous abortion; CTS, *Chlamydia trachomatis* serostatus.

1 Cases are those individuals with defined signs or symptoms; controls are individuals without signs or symptoms.

2 Population prevalance and OR calculation of *M. genitalium* infection with infertility were evaluated using a serological assay targeting MgPa.

3 *M. genitalium* was not detected by PCR in any of 210 available specimens; *M. genitalium* seroprevalence was 7% among 212 tested women.

4 Women with CT-positive ELISAs were excluded.

In contrast, investigations into *M. genitalium* as a cause of infertility have consistently shown a strong correlation. Two Danish studies have found a significant association between women with *M. genitalium*–specific serum antibodies and laparoscopically confirmed tubal infertility ([17], [24]; Table 4). Exclusion of women with prior *C. trachomatis* infection still resulted in significant association between *M. genitalium* and infertility ([24]; Table 4). In one study, PCR detection of *M. genitalium* was attempted from endocervical swabs with no success, suggesting that previous *M. genitalium* infection might cause permanent damage to the oviduct, or that endocervical swabs are ineffective for detecting upper genital tract infection. However, in a recent study of Polish women by Grzesko and colleagues, *M. genitalium* was detected by PCR more often in cervical swabs from infertile patients compared to healthy, fertile women [37], suggesting that endocervical swabs can predict upper tract infection. It is important to note that NAAT studies make associations between current infection and infertility, while serological studies determine associations with prior *M. genitalium* exposure. Because tubal scarring can result in long-term infertility, serological studies probably best address whether *M. genitalium* is a cause of tubal-factor infertility and can be useful in determining recent or long-term infections (e.g., IgM versus IgG antibodies).

Experimental animal models have also provided evidence that *M. genitalium* can colonize upper reproductive tract tissues, leading to salpingitis or endometritis [61]–[63]. Thus, it is evident that *M. genitalium* could be an independent cause of tubal factor infertility. Importantly, however, the few studies to date have been relatively small in size and longitudinal studies would be of tremendous benefit for understanding this complex condition whereby prior infection can lead to long-term sequelae.

## Recommended Treatment of *M. genitalium* and Important Considerations

Evaluation of *M. genitalium* treatment efficacy has been a subject of obvious importance but conclusive recommendations are lacking largely because, to date, only a single randomized controlled clinical trial has been reported [64]. In this trial, a single 1-g dose of azithromycin was more effective than 7-day, multi-dose doxycycline for eradication of *M. genitalium* infection in men. In patients diagnosed with PID, the US Centers for Disease Control and Prevention guidelines recommend therapy with ceftriaxone plus doxycycline or cefoxitin and probenecid plus doxycycline [65]. These treatments are primarily targeted towards *C. trachomatis* and *N. gonorrhoeae*, to which less than half of PID cases can be attributed [66]. Importantly, several reports suggest these treatment regimens would be ineffective for eradicating *M. genitalium*, as male and female genital infections persist in a significant proportion of patients treated with tetracyclines [67]–[71] or levofloxacin [68], [72].

Using azithromycin (1 g; single dose), clinical cure rates are only between 79% and 87% for *M. genitalium*–positive male and female patients, leaving a significant subset of patients with persistent urogenital tract infections [29], [64], [67], [73]. Extended 5-day regimens of azithromycin therapy increase cure rates to 96% after doxycycline treatment failure [67], and additional randomized trials are now required to determine the optimum dosage and regimen. If patients fail extended azithromycin therapy, moxifloxacin is the only available antibiotic with a successful rate of cure [73] and should be used only with patients failing other therapies. Successful treatment of *M. genitalium* infection in female patients is of particular importance because prolonged inflammation at upper genital tract sites might lead to significant reproductive tract morbidity and infertility [74]. In women, treatment must be effective for both lower and upper genital tract infection.

## Conclusions and Implications for Future Research

Following the firm establishment that *M. genitalium* causes NGU in men and is a cause of STI, many studies have now found significant associations with lower and upper reproductive tract disease in women. Taken together, *M. genitalium* should be considered an etiologic agent of cervical inflammation and upper tract disease syndromes, including PID and infertility. Importantly, additional studies with defined diagnostic criteria will be required to fully understand the relationship between *M. genitalium* and cervicitis. A systematic review and meta-analysis would be of significant benefit for defining the associations of *M. genitalium* infection with reproductive tract disease in women.

Although not addressed in this review, *M. genitalium* likely maintains persistent infection through intracellular survival in mucosal epithelial cells [75], [76] resulting in inflammation [75], [77]. The observed correlations between *M. genitalium* reproductive tract infection and HIV-1 (reviewed in [78]) may be explained by long-term inflammation elicited by *M. genitalium* infection; these associations are likely of particular importance considering the enormous public health burden of HIV infections worldwide. Therefore, continued research will be important to understand the dynamics of persistent HIV-1 and *M. genitalium* co-infections of vaginal and cervical tissues, particularly when dissecting clinical correlates with disease.

We still have much to learn about reproductive tract infections from both a clinical and basic science standpoint. Overall, *M. genitalium* appears to be a highly prevalent sexually transmitted bacterial pathogen that, if not diagnosed and the patient treated appropriately, can cause persistent urogenital inflammation in men and women and increase the risk of HIV transmission and infection. Continued investigation of *M. genitalium*'s role in sexually transmitted disease will be pivotal for understanding the complex and dynamic inflammatory syndromes of the female reproductive tract.

## Supporting Information

Table S1 Comprehensive summary of published studies of women where urogenital *M. genitalium* prevalence was determined using a nucleic acid amplification test (NAAT).
(0.18 MB DOC)

## References

[1] World Health Organization (2007). Sexually transmitted infections fact sheet.. http://www.who.int/mediacentre/factsheets/fs110/en/.

[2] Manhart LE, Holmes KK, Hughes JP, Houston LS, Totten PA (2007). *Mycoplasma genitalium* among young adults in the United States: an emerging sexually transmitted infection.. Am J Public Health.

[3] Tully JG, Taylor-Robinson D, Cole RM, Rose DL (1981). A newly discovered mycoplasma in the human urogenital tract.. Lancet.

[4] Jensen JS (2004). *Mycoplasma genitalium*: the aetiological agent of urethritis and other sexually transmitted diseases.. J Eur Acad Dermatol Venereol.

[5] Martin DH (2008). Nongonococcal urethritis: new views through the prism of modern molecular microbiology.. Curr Infect Dis Rep.

[6] Jensen JS, Uldum SA, Sondergard-Andersen J, Vuust J, Lind K (1991). Polymerase chain reaction for detection of *Mycoplasma genitalium* in clinical samples.. J Clin Microbiol.

[7] Palmer HM, Gilroy CB, Claydon EJ, Taylor-Robinson D (1991). Detection of *Mycoplasma genitalium* in the genitourinary tract of women by the polymerase chain reaction.. Int J STD AIDS.

[8] Anagrius C, Lore B, Jensen JS (2005). *Mycoplasma genitalium*: prevalence, clinical significance, and transmission.. Sex Transm Infect.

[9] Falk L, Fredlund H, Jensen JS (2004). Symptomatic urethritis is more prevalent in men infected with *Mycoplasma genitalium* than with *Chlamydia trachomatis*.. Sex Transm Infect.

[10] Falk L, Fredlund H, Jensen JS (2005). Signs and symptoms of urethritis and cervicitis among women with or without *Mycoplasma genitalium* or *Chlamydia trachomatis* infection.. Sex Transm Infect.

[11] Hjorth SV, Bjornelius E, Lidbrink P, Falk L, Dohn B (2006). Sequence-based typing of *Mycoplasma genitalium* reveals sexual transmission.. J Clin Microbiol.

[12] Keane FE, Thomas BJ, Gilroy CB, Renton A, Taylor-Robinson D (2000). The association of *Chlamydia trachomatis* and *Mycoplasma genitalium* with non-gonococcal urethritis: observations on heterosexual men and their female partners.. Int J STD AIDS.

[13] Thurman AR, Musatovova O, Perdue S, Shain RN, Baseman JG (2010). *Mycoplasma genitalium* symptoms, concordance and treatment in high-risk sexual dyads.. Int J STD AIDS.

[14] Tosh AK, Van Der Pol B, Fortenberry JD, Williams JA, Katz BP (2007). *Mycoplasma genitalium* among adolescent women and their partners.. J Adolesc Health.

[15] Ma L, Taylor S, Jensen JS, Myers L, Lillis R (2008). Short tandem repeat sequences in the *Mycoplasma genitalium* genome and their use in a multilocus genotyping system.. BMC Microbiol.

[16] Andersen B, Sokolowski I, Ostergaard L, Kjolseth Moller J, Olesen F (2007). *Mycoplasma genitalium*: prevalence and behavioural risk factors in the general population.. Sex Transm Infect.

[17] Clausen HF, Fedder J, Drasbek M, Nielsen PK, Toft B (2001). Serological investigation of *Mycoplasma genitalium* in infertile women.. Hum Reprod.

[18] Edwards RK, Ferguson RJ, Reyes L, Brown M, Theriaque DW (2006). Assessing the relationship between preterm delivery and various microorganisms recovered from the lower genital tract.. J Matern Fetal Neonatal Med.

[19] Ghebremichael M, Paintsil E, Larsen U (2009). Alcohol abuse, sexual risk behaviors, and sexually transmitted infections in women in Moshi urban district, northern Tanzania.. Sex Transm Dis.

[20] Hitti J, Garcia P, Totten P, Paul K, Astete S (2010). Correlates of cervical *Mycoplasma genitalium* and risk of preterm birth among Peruvian women.. Sex Transm Dis.

[21] Oakeshott P, Hay P, Taylor-Robinson D, Hay S, Dohn B (2004). Prevalence of *Mycoplasma genitalium* in early pregnancy and relationship between its presence and pregnancy outcome.. BJOG.

[22] Olsen B, Lan PT, Stalsby Lundborg C, Khang TH, Unemo M (2009). Population-based assessment of *Mycoplasma genitalium* in Vietnam–low prevalence among married women of reproductive age in a rural area.. J Eur Acad Dermatol Venereol.

[23] Rahman S, Garland S, Currie M, Tabrizi SN, Rahman M (2008). Prevalence of *Mycoplasma genitalium* in health clinic attendees complaining of vaginal discharge in Bangladesh.. Int J STD AIDS.

[24] Svenstrup HF, Fedder J, Kristoffersen SE, Trolle B, Birkelund S (2008). *Mycoplasma genitalium*, *Chlamydia trachomatis*, and tubal factor infertility–a prospective study.. Fertil Steril.

[25] Arraiz RN, Colina Ch S, Marcucci JR, Rondon GN, Reyes SF (2008). *Mycoplasma genitalium* detection and correlation with clinical manifestations in population of the Zulia State, Venezuela.. Rev Chilena Infectol.

[26] Baczynska A, Hvid M, Lamy P, Birkelund S, Christiansen G (2008). Prevalence of *Mycoplasma genitalium*, *Mycoplasma hominis* and *Chlamydia trachomatis* among Danish patients requesting abortion.. Syst Biol Reprod Med.

[27] Bjartling C, Osser S, Persson K (2010). The association between *Mycoplasma genitalium* and pelvic inflammatory disease after termination of pregnancy.. BJOG.

[28] Blanchard A, Hamrick W, Duffy L, Baldus K, Cassell GH (1993). Use of the polymerase chain reaction for detection of *Mycoplasma fermentans* and *Mycoplasma genitalium* in the urogenital tract and amniotic fluid.. Clin Infect Dis.

[29] Bradshaw CS, Chen MY, Fairley CK (2008). Persistence of *Mycoplasma genitalium* following azithromycin therapy.. PLoS ONE.

[30] Casin I, Vexiau-Robert D, De La Salmoniere P, Eche A, Grandry B (2002). High prevalence of *Mycoplasma genitalium* in the lower genitourinary tract of women attending a sexually transmitted disease clinic in Paris, France.. Sex Transm Dis.

[31] Cohen CR, Manhart LE, Bukusi EA, Astete S, Brunham RC (2002). Association between *Mycoplasma genitalium* and acute endometritis.. Lancet.

[32] Cohen CR, Mugo NR, Astete SG, Odondo R, Manhart LE (2005). Detection of *Mycoplasma genitalium* in women with laparoscopically diagnosed acute salpingitis.. Sex Transm Infect.

[33] Cohen CR, Nosek M, Meier A, Astete SG, Iverson-Cabral S (2007). *Mycoplasma genitalium* infection and persistence in a cohort of female sex workers in Nairobi, Kenya.. Sex Transm Dis.

[34] de Barbeyrac B, Bernet-Poggi C, Febrer F, Renaudin H, Dupon M (1993). Detection of *Mycoplasma pneumoniae* and *Mycoplasma genitalium* in clinical samples by polymerase chain reaction.. Clin Infect Dis.

[35] Edberg A, Jurstrand M, Johansson E, Wikander E, Hoog A (2008). A comparative study of three different PCR assays for detection of *Mycoplasma genitalium* in urogenital specimens from men and women.. J Med Microbiol.

[36] Gaydos C, Maldeis NE, Hardick A, Hardick J, Quinn TC (2009). *Mycoplasma genitalium* as a contributor to the multiple etiologies of cervicitis in women attending sexually transmitted disease clinics.. Sex Transm Dis.

[37] Grzesko J, Elias M, Maczynska B, Kasprzykowska U, Tlaczala M (2009). Occurrence of *Mycoplasma genitalium* in fertile and infertile women.. Fertil Steril.

[38] Haggerty CL, Totten PA, Astete SG, Ness RB (2006). *Mycoplasma genitalium* among women with nongonococcal, nonchlamydial pelvic inflammatory disease.. Infect Dis Obstet Gynecol.

[39] Haggerty CL, Totten PA, Astete SG, Lee S, Hoferka SL (2008). Failure of cefoxitin and doxycycline to eradicate endometrial *Mycoplasma genitalium* and the consequence for clinical cure of pelvic inflammatory disease.. Sex Transm Infect.

[40] Hogdahl M, Kihlstrom E (2007). Leucocyte esterase testing of first-voided urine and urethral and cervical smears to identify *Mycoplasma genitalium*-infected men and women.. Int J STD AIDS.

[41] Huppert JS, Mortensen JE, Reed JL, Kahn JA, Rich KD (2008). *Mycoplasma genitalium* detected by transcription-mediated amplification is associated with *Chlamydia trachomatis* in adolescent women.. Sex Transm Dis.

[42] Jurstrand M, Jensen JS, Magnuson A, Kamwendo F, Fredlund H (2007). A serological study of the role of *Mycoplasma genitalium* in pelvic inflammatory disease and ectopic pregnancy.. Sex Transm Infect.

[43] Korte JE, Baseman JB, Cagle MP, Herrera C, Piper JM (2006). Cervicitis and genitourinary symptoms in women culture positive for *Mycoplasma genitalium*.. Am J Reprod Immunol.

[44] Labbe AC, Frost E, Deslandes S, Mendonca AP, Alves AC (2002). *Mycoplasma genitalium* is not associated with adverse outcomes of pregnancy in Guinea-Bissau.. Sex Transm Infect.

[45] Lawton BA, Rose SB, Bromhead C, Gaitanos LA, MacDonald EJ (2008). High prevalence of *Mycoplasma genitalium* in women presenting for termination of pregnancy.. Contraception.

[46] Manhart LE, Critchlow CW, Holmes KK, Dutro SM, Eschenbach DA (2003). Mucopurulent cervicitis and *Mycoplasma genitalium*.. J Infect Dis.

[47] Manhart LE, Mostad SB, Baeten JM, Astete SG, Mandaliya K (2008). High *Mycoplasma genitalium* organism burden is associated with shedding of HIV-1 DNA from the cervix.. J Infect Dis.

[48] Mellenius H, Boman J, Lundqvist EN, Jensen JS (2005). *Mycoplasma genitalium* should be suspected in unspecific urethritis and cervicitis. A study from Vasterbotten confirms the high prevalence of the bacteria.. Lakartidningen.

[49] Moi H, Reinton N, Moghaddam A (2009). *Mycoplasma genitalium* in women with lower genital tract inflammation.. Sex Transm Infect.

[50] Musatovova O, Baseman JB (2009). Analysis identifying common and distinct sequences among Texas clinical strains of *Mycoplasma genitalium*.. J Clin Microbiol.

[51] Pepin J, Labbe AC, Khonde N, Deslandes S, Alary M (2005). *Mycoplasma genitalium*: an organism commonly associated with cervicitis among west African sex workers.. Sex Transm Infect.

[52] Ross JD, Brown L, Saunders P, Alexander S (2009). *Mycoplasma genitalium* in asymptomatic patients: implications for screening.. Sex Transm Infect.

[53] Short VL, Jensen JS, Nelson DB, Murray PJ, Ness RB (2010). *Mycoplasma genitalium* among young, urban pregnant women.. Infect Dis Obstet Gynecol.

[54] Simms I, Eastick K, Mallinson H, Thomas K, Gokhale R (2003). Associations between *Mycoplasma genitalium*, *Chlamydia trachomatis* and pelvic inflammatory disease.. J Clin Pathol.

[55] Tsunoe H, Tanaka M, Nakayama H, Sano M, Nakamura G (2000). High prevalence of *Chlamydia trachomatis*, *Neisseria gonorrhoeae* and *Mycoplasma genitalium* in female commercial sex workers in Japan.. Int J STD AIDS.

[56] Uno M, Deguchi T, Komeda H, Hayasaki M, Iida M (1997). *Mycoplasma genitalium* in the cervices of Japanese women.. Sex Transm Dis.

[57] Spence D, Melville C (2007). Vaginal discharge.. BMJ.

[58] Marrazzo JM, Martin DH (2007). Management of women with cervicitis.. Clin Infect Dis.

[59] Moller BR, Taylor-Robinson D, Furr PM (1984). Serological evidence implicating *Mycoplasma genitalium* in pelvic inflammatory disease.. Lancet.

[60] Westrom L (1975). Effect of acute pelvic inflammatory disease on fertility.. Am J Obstet Gynecol.

[61] McGowin CL, Spagnuolo RA, Pyles RB (2010). *Mycoplasma genitalium* rapidly disseminates to the upper reproductive tracts and knees of female mice following vaginal inoculation.. Infect Immun.

[62] Moller BR, Taylor-Robinson D, Furr PM, Freundt EA (1985). Acute upper genital-tract disease in female monkeys provoked experimentally by *Mycoplasma genitalium*.. Br J Exp Pathol.

[63] Taylor-Robinson D, Furr PM, Tully JG, Barile MF, Moller BR (1987). Animal models of *Mycoplasma genitalium* urogenital infection.. Isr J Med Sci.

[64] Mena LA, Mroczkowski TF, Nsuami M, Martin DH (2009). A randomized comparison of azithromycin and doxycycline for the treatment of *Mycoplasma genitalium*-positive urethritis in men.. Clin Infect Dis.

[65] Workowski KA, Berman S (2010). Sexually transmitted diseases treatment guidelines, 2010.. MMWR Recomm Rep.

[66] Ness RB, Soper DE, Holley RL, Peipert J, Randall H (2002). Effectiveness of inpatient and outpatient treatment strategies for women with pelvic inflammatory disease: results from the Pelvic Inflammatory Disease Evaluation and Clinical Health (PEACH) Randomized Trial.. Am J Obstet Gynecol.

[67] Bjornelius E, Anagrius C, Bojs G, Carlberg H, Johannisson G (2008). Antibiotic treatment of symptomatic *Mycoplasma genitalium* infection in Scandinavia: a controlled clinical trial.. Sex Transm Infect.

[68] Deguchi T, Yoshida T, Yokoi S, Ito M, Tamaki M (2002). Longitudinal quantitative detection by real-time PCR of *Mycoplasma genitalium* in first-pass urine of men with recurrent nongonococcal urethritis.. J Clin Microbiol.

[69] Falk L, Fredlund H, Jensen JS (2003). Tetracycline treatment does not eradicate *Mycoplasma genitalium*.. Sex Transm Infect.

[70] Horner PJ, Gilroy CB, Thomas BJ, Naidoo RO, Taylor-Robinson D (1993). Association of *Mycoplasma genitalium* with acute non-gonococcal urethritis.. Lancet.

[71] Wikstrom A, Jensen JS (2006). *Mycoplasma genitalium*: a common cause of persistent urethritis among men treated with doxycycline.. Sex Transm Infect.

[72] Maeda SI, Tamaki M, Kojima K, Yoshida T, Ishiko H (2001). Association of *Mycoplasma genitalium* persistence in the urethra with recurrence of nongonococcal urethritis.. Sex Transm Dis.

[73] Jernberg E, Moghaddam A, Moi H (2008). Azithromycin and moxifloxacin for microbiological cure of *Mycoplasma genitalium* infection: an open study.. Int J STD AIDS.

[74] Quayle AJ (2002). The innate and early immune response to pathogen challenge in the female genital tract and the pivotal role of epithelial cells.. J Reprod Immunol.

[75] McGowin CL, Popov VL, Pyles RB (2009). Intracellular *Mycoplasma genitalium* infection of human vaginal and cervical epithelial cells elicits distinct patterns of inflammatory cytokine secretion and provides a possible survival niche against macrophage-mediated killing.. BMC Microbiol.

[76] Ueno PM, Timenetsky J, Centonze VE, Wewer JJ, Cagle M (2008). Interaction of *Mycoplasma genitalium* with host cells: evidence for nuclear localization.. Microbiology.

[77] McGowin CL, Ma L, Martin DH, Pyles RB (2009). *Mycoplasma genitalium*-encoded MG309 activates NF-kappaB via Toll-like receptors 2 and 6 to elicit proinflammatory cytokine secretion from human genital epithelial cells.. Infect Immun.

[78] Napierala Mavedzenge S, Weiss HA (2009). Association of *Mycoplasma genitalium* and HIV infection: a systematic review and meta-analysis.. AIDS.

