# Supplementary material for: Mycoplasma genitalium: An Emerging Cause of Sexually Transmitted Disease in Women
Source: PLoS Pathog. 2011 May 26;7(5):e1001324. doi: 10.1371/journal.ppat.1001324 (PMC3102684; doi:10.1371/journal.ppat.1001324)
Supplement: Table S1 — Comprehensive summary of published studies of women where urogenital M. genitalium prevalence was determined using a nucleic acid amplification test (NAAT). (0.18 MB DOC) [file ppat.1001324.s001.doc]

**Table S1. Comprehensive summary of published studies of women where urogenital *M. genitalium* prevalence was determined using a nucleic acid amplification test (NAAT).**

| **Reference, clinical setting and any specific criteria for enrollment in study** | **No. tested** | **MG prevalence (%)** | **Specimen type for MG detection** | **Diagnostic method** |
| --- | --- | --- | --- | --- |
| **STUDIES OF HIGH-RISK POPULATIONS** |  |  |  |  |
| **Anagrius, 2005** [8] (STI clinic, Sweden) | 445 | 6.3 | ES and US | PCR |
| **Arraiz, 2008** [25] (Private Gyn clinic; genitourinary signs, Venezuela) | 172^1^ | 7.6^1^ | ES | PCR |
| **Baczynska, 2008** [26] (Hospital Ob/Gyn, requesting TOP, Denmark) | 102 | 1.0 | ES | RTPCR |
| **Bertille de Barbeyrac, 1993** [34] (STI clinic, France) | 55 | 0 | US or ES | Cult/PCR+SB |
| **Bjartling, 2010** [27] (Hospital Ob/Gyn, requesting TOP, Sweden) | 2079 | 2.5 | Urine and/or VS, ES | PCR |
| **Blanchard, 1993** [28] (STI clinic or hospital Ob/Gyn controls, cervicitis, USA) | 282 | 1.1 | US, ES or AF | PCR |
| **Bradshaw, 2008** [29] (STI clinic, urogenital symptoms, Australia) | 313 | 9.6 | FVU or ES | RTPCR |
| **Casin, 2002** [30] (STI clinic, vaginal discharge, France) | 170 | 38.2^2^ | ES, VS and/or US | PCR |
| **Cohen, 2002** [31] (STI clinic, pelvic pain >14d, Kenya) | 115 | 8.7 | ES or EMB | PCR |
| **Cohen, 2005** [32] (Hospital Ob/Gyn, PID & confirmed salpingitis, Kenya) | 123 | 7.3 | ES, EMB, TU | PCR |
| **Cohen, 2007** [33] (Public health clinic, CSW, Kenya) | 255 | 15.7 | ES and EMB | PCR |
| **Edberg, 2008** [35] (STI clinic, partner with *M. genitalium*, Sweden) | 298 | 7.7 | FVU, ES and/or US | RTPCR |
| **Falk, 2005** [10] (STI clinic or cancer screen controls, Sweden) | 520 | 5.0 | FVU and ES | PCR |
| **Gaydos, 2009** [36] (STI clinic, USA) | 322 | 19.3 | VS | RTPCR and TMA |
| **Grzesko, 2009** [37] (Hospital Ob/Gyn patients, confirmed infertility, Poland) | 74 | 14.9 | ES | PCR |
| **Haggerty, 2006** [38] (ER, Ob/Gyn, STI clinic, or primary health, NGNC EM or cervicitis, USA) | 50^3^ | 14.0^3^ | ES and EMB | PCR+SB |
| **Haggerty, 2008** [39] (ER, Ob/Gyn, STI, primary health clinic, endometritis, USA) | 586 | 15.0 | ES and EMB | PCR+SB |
| **Hogdahl, 2007** [40] (STI clinic, Sweden) | 417 | 6.5 | FVU | RTPCR |
| **Huppert, 2008** [41] (THC or ER, genital signs or high-risk behavior, USA) | 331 | 22.3 | VS | TMA |
| **Jensen, 1991** [6] (Primary health or STI clinic, Denmark) | 74 | 6.8 | ES | PCR+SB |
| **Jurstrand, 2007** [42] (Ob/Gyn, PID or ectopic pregnancy, Sweden) | 521^4^ | 16.1^4^ | N/A | Serological |
| **Korte, 2006** [43] (PHC, STI at enrollment, USA) | 674^3^ | 42.0^3^ | VS and ES | Cult/PCR+SB |
| **Labbe, 2002** [44] (Hospital Ob/Gyn, pre-term or controls at term, Guinea-Bassau) | 1014 | 6.2 | ES | PCR |
| **Lawton, 2008** [45] (Hospital Ob/Gyn, requesting TOP, New Zealand) | 300 | 8.6 | VS | RTPCR |
| **Manhart, 2003** [46] (STI clinic, USA) | 719 | 7.0 | CS | PCR+SB |
| **Manhart, 2008** [47] (STI clinic, HIV+, 72% CSW, Kenya) | 303 | 17.2 | ES | PCR+DIG |
| **Mellenius, 2005** [48] (STI clinic, Sweden) | 340 | 3.8 | ES | RTPCR |
| **Moi, 2009** [49] (STI clinic, genital signs or high-risk behavior, Norway) | 7646 | 4.0 | FVU and/or ES | RTPCR |
| **Musatovova, 2009** [50] (PHC, non-viral STI at enrollment, USA) | 268^3^ | 16.8^3^ | Urine, VS and ES | RTPCR |
| **Palmer, 1991** [7] (STI clinic, UK) | 57 | 19.3 | ES, VS and/or US | PCR |
| **Pepin, 2005** [51] (STI clinic, CSW, Benin/Ghana) | 826 | 26.3 | ES | PCR |
| **Ross, 2009** [52] (STI clinic, asymptomatic, UK) | 138 | 6.5 | ES or VS | RTPCR |
| **Short, 2010** [53] (Pregnant ER patients <22wks gestation, USA) | 216 | 5.6 | Urine | RTPCR |
| **Simms, 2003** [54] (STI clinic, Hospital Ob/Gyn, primary health clinic for controls, UK) | 82 | 7.3 | ES | RTPCR |
| **Thurman, 2010** [13] (PHC, STI at enrollment, USA) | 590^5^ | NC^5^ | Urine and ES | RTPCR |
| **Tsunoe, 2000** [55] (CSW attending STI clinic or pregnant controls, Japan) | 174^6^ | 12.6^6^ | ES | PCR |
| **Uno, 1997** [56] (Hospital Ob/Gyn patients, Japan) | 200 | 4.5 | ES | PCR+SB |
| **Total no. of studies of high-risk populations: 37** | **18748** | **7.3%** |  |  |
|  |  |  |  |  |
| **STUDIES OF LOW-RISK POPULATIONS** |  |  |  |  |
|  |  |  |  |  |
| **Andersen, 2007** [16] (In home testing, randomized population screen, Denmark) | 921 | 2.3 | VP | RTPCR |
| **Clausen, 2001** [17] (IVF clinic, confirmed infertility, Denmark) | 308^4^ | 13.0^4^ | N/A | Serological |
| **Edwards, 2006** [18] **(**Hospital Ob/Gyn, signs/symptoms of preterm labor, USA) | 134 | 20.2 | VL | PCR |
| **Ghebremichael, 2009** [19] (PHC, randomized population screen, Tanzania) | 1440 | 3.3 | Urine | RTPCR |
| **Hitti, 2010** [20] (Hospital Ob/Gyn, delivering pre-term or controls at term, Peru) | 1328 | 3.1 | ES | TMA |
| **Manhart, 2007** [2] (In home testing, randomized population screen, USA) | 1714 | 0.8 | FVU | PCR |
| **Oakeshott, 2004** [21] (Primary health or family planning clinic, pregnant, UK) | 915 | 0.7 | FVU | PCR |
| **Olsen, 2009** [22] (PHC, healthy married women, Vietnam) | 990 | 0.8 | ES | RTPCR |
| **Rahman, 2008** [23] (Primary health care, vaginal discharge, Bangladesh) | 399 | 0.8 | ES | RTPCR |
| **Svenstrup, 2008** [24] (Fertility clinic, confirmed infertility, Denmark) | 210 | 0 | ES | RTPCR |
| **Tosh, 2007** [14] (Primary health care clinic, USA) | 383 | 0.8 | VS | PCR+DIG |
|  |  |  |  |  |
| **Total no. of studies of low-risk populations: 11** | **8434** | **2.0%** |  |  |
|  |  |  |  |  |
| **Total no. of studies of high- and low-risk populations: 48** | **27182** | **5.6%** |  |  |

STI, sexually transmitted infection; PHC, public health clinic; VS, vaginal swab; ES, endocervical swab; FVU, first void urine; EMB, endometrial biopsy; TU, tubal fluid or biopsy; US, urethral swab; AF, amniotic fluid; CS, cervical secretions; VP, vaginal pipette; RTPCR, Real-time PCR; PCR+SB, PCR with southern blot confirmation; TMA, transcription mediated amplification; PCR+DIG, PCR with specific digoxigenin probe for confirmation; IVF, in vitro fertilization; NGNC EM, non-gonococcal, non-chlamydial endometritis; TOP, termination of pregnancy; CSW, commercial sex workers; NAAT, nucleic acid amplification test; NC, point prevalence at enrollment not calculable from presented data; VL, vaginal lavage

^1^Excluded patients with CT or human papilloma virus

^2^Prevalence calculated from PCR positive results from any of up to 3 sites sampled at enrollment

^3^Not included in overall prevalence calculation; patient population also represented in another study

^4^Based on serological assay, not included in overall NAAT prevalence calculation

^5^Not included in overall prevalence calculation; point prevalence at enrollment not calculable from presented data

^6^Among only STI clinic attendees
